# Measured Glomerular Filtration Rate in Live Related Kidney Donors Three Months Post-Kidney Donation: A Single-Center Experience From Western India

**DOI:** 10.7759/cureus.45103

**Published:** 2023-09-12

**Authors:** Abhijit S Chavan, Charan B Bale, Pavan S Wakhare, Nilesh Shinde, Akshay R Kulkarni, Atul D Sajgure, Tushar A Dighe

**Affiliations:** 1 Nephrology, Dr. D. Y. Patil Medical College, Hospital & Research Centre, Dr. D. Y. Patil Vidyapeeth, Pune, IND

**Keywords:** donor evaluation, diethylene triamine pentaacetic acid (dtpa) renal scan, kidney donors, glomerular filtration rate (gfr), living donor nephrectomy

## Abstract

Background

Glomerular filtration rate (GFR) estimation is pivotal in the evaluation of kidney donors. There are various methods available for assessing GFR, but there has been a lack of consensus on the measurement of GFR and the frequency of renal evaluation after kidney donation. Our study aims to analyze the measured GFR (m-GFR) before and three months after kidney donation and note the compensatory abilities of the remnant kidney in live related kidney donors.

Methods

This prospective observational study was conducted at the Department of Nephrology, Dr. D. Y. Patil Medical College, Hospital & Research Centre, Pune, from April 2021 to December 2022. The study included 30 donors from both genders aged between 23 and 73 years. The measured GFR was calculated using a technetium-99m diethylene triamine pentaacetic acid (Tc-99m DTPA) scan. We analyzed donor characteristics and various parameters that included demography, anthropometry, blood pressure, and serum creatinine and measured GFR (m-GFR) using a Tc-99m DTPA scan, which was compared before and three months after donor nephrectomy.

Results

Of the 30 donors, 25 (83.3%) were females and five (16.7%) were males. The mean age of donors was 49.23 ± 12.29 years. The mean body mass index (BMI) was noted to be 24.73 ± 5.58 kg/m^2^, whereas the mean body surface area (BSA) was 1.59 ± 0.12 m^2^. In terms of the measured GFR by DTPA scan, pre-donation and post-donation, the average GFR for our population was 103.83 ± 10.07 mL/minute/1.73 m^2 ^and^ ^60.47±6.57 mL/minute/1.73 m^2^, respectively. The mean measured GFR of remnant kidney increased by 9.21 ± 4.39 mL/minute/1.73 m^2^ in 28 donors, while two donors had a fall in the mean measured GFR by 6.8 ± 1.69 mL/minute/1.73 m^2^.

Conclusions

To safeguard donor health, accurate measurement of GFR at various timelines after kidney donation should be considered as there are various limitations associated with the use of serum creatinine-based GFR estimating equations for solitary kidneys. However, long-term studies are required to analyze the changes in GFR after nephrectomy and determine the adequacy of compensatory changes in the remnant kidney post-kidney donation.

## Introduction

Living kidney donor transplantation has been considered to have superior outcomes as compared to deceased donor transplantation for patients with end-stage kidney disease (ESKD) [1]. From 2013 to 2018, there were 32,584 living donor kidney transplants and 5,748 deceased donor kidney transplants in India, signifying a greater trend of organ donation from living donors as compared to deceased donation [2]. To undergo a living kidney donation, an individual must undergo a plethora of tests and medical evaluations to be considered fit for kidney donation. Among these, glomerular filtration rate (GFR) estimation is pivotal in the evaluation of potential kidney donors and lies at the core of kidney donor assessment protocols.

Although many studies have emphasized that there is a lower likelihood of developing chronic kidney disease after living kidney donation [3], minimizing the risk of end-stage kidney disease in donors is of utmost importance for maintaining optimal donor health. Recent studies have reported a three- to fivefold increased relative risk of kidney failure after a unilateral nephrectomy, although the absolute risk remained minimal [4-6]. In today’s scenario, many prospective kidney donors have comorbidities and fall into an elderly age group [7]. Renal insufficiency of about 0.2%-0.5% has been reported after donor nephrectomy [8]. This makes it imperative that they undergo a thorough assessment after unilateral nephrectomy as well. The selection and medical care of living kidney donors can be improved with a better understanding of the risk factors to prevent ESKD in living kidney donors [9]. After unilateral nephrectomy, the remnant kidney undergoes functional changes, which include hemodynamic alterations and glomerular hypertrophy [10]. It is important to analyze these changes and look for adequacy of the renal function considering the functional renal loss that occurred after nephrectomy.

Although there are various methods available for measuring GFR, there has been a lack of consensus on the assessment of GFR after kidney donation, its method of measurement, and the frequency of renal evaluation. In this study, we aimed to measure the GFR using a technetium-99m diethylene triamine pentaacetic acid (Tc-99m DTPA) scan done up to 14 weeks pre-nephrectomy and 11-13 weeks post-nephrectomy, and changes in the measured GFR (m-GFR) were analyzed before and three months after donor nephrectomy.

A part of this study was previously presented as a poster at the American Society of Nephrology Kidney Week 2022 on November 5, 2022.

## Materials and methods

This was a prospective observational study of a cohort of 30 donors who, after appropriate medical and surgical evaluation, underwent donor nephrectomy for living related kidney transplants between April 2021 and December 2022 at Dr. D. Y. Patil Medical College, Hospital & Research Centre, Pune, India. For the study, an institutional ethical committee approval was obtained with reference number IESC/S.SP/2020/03. All participants had to sign a written informed consent in accordance with the ethical standards and guidelines. All the healthy live related kidney donors were near relatives of the recipients, which included parents, siblings, and spouses, aged between 23 and 73 years and with a body mass index (BMI) of less than 35 kg/m^2^. Donors with ages more than 75 years, uncontrolled hypertension, diabetes mellitus, microalbuminuria, and measured GFR by Tc-99m DTPA scan of less than 80 mL/minute/1.73 m^2^ were excluded from the study. A comprehensive medical history along with demographic parameters and detailed clinical examination that encompassed height, weight, BMI (kg/m^2^), and body surface area (BSA) (m^2^) calculated using the Du Bois method [11] were noted. Office blood pressure measurements were measured after 10 minutes of rest using the automated oscillometric device Omron (HEM-7121 model) (Kyoto, Japan), on the nondominant arm, with a suitable-sized cuff, with the elbow rested in the sitting position. Two readings were recorded over five minutes, and their mean was taken. The validity of a recording and definition of hypertension were in accordance with the Eighth Joint National Committee (JNC 8) guidelines [12].

Further evaluation of all donors for this study included serum creatinine and GFR measurements by Tc-99m DTPA scan done up to 14 weeks prior to donor nephrectomy, followed by 11-13 weeks after donor nephrectomy. Serum creatinine was measured by alkaline picrate kinetic method using Siemens Dimension EXL 200 Integrated Chemistry System (Munich, Germany). Prior to the DTPA scan, all subjects were ensured to have adequate hydration. The subjects were then injected intravenously with 228 MBq (4 mCi) Tc-99m DTPA, and dynamic images were acquired at two seconds per frame for one minute and subsequently at a rate of 60 seconds/frame for 20 minutes using the Gates protocol. Subsequently, GFR values were calculated after standardization of GFR as per standard body surface area of 1.73 m^2^.

Statistical analysis

Data was collected using a prestructured questionnaire entered in Microsoft Excel 2019 (Microsoft Corporation, Redmond, WA, USA). Variables such as age, weight, BMI, GFR, blood pressure readings, and serum creatinine were expressed as mean and standard deviation (SD), while qualitative variables such as comorbidities and gender were represented by frequencies and percentages. The Student’s paired t-test was used for comparison of continuous variables. Statistical tests for data analysis were applied using Statistical Package for the Social Sciences (SPSS) for Windows version 22.0 (IBM SPSS Statistics, Armonk, NY, USA). Statistical significance was considered at p < 0.05 at a 95% confidence interval.

## Results

We analyzed 30 donors, which included 25 females (83.33%) and five males (16.66%). Multiple parameters including demography, anthropometry, pre- and post-nephrectomy blood pressure, serum creatinine, and measured GFR were studied. The age of all donors ranged from 23 to 73 years, with a mean age of 49.23 ± 12.29 years. The weight, BMI, and BSA ranged from 43 to 93 kg, 17.3 to 33.3 kg/m^2^, and 1.39 to 1.85 m^2^, respectively. The mean weight, BMI, and BSA recorded were 60.72 kg, 24.73 kg/m^2^, and 1.59 m^2^, respectively (Table 1). Out of 30 donors, seven (35%) had hypertension, three (15%) had hypothyroidism, two (6.66%) were obese, and none had diabetes mellitus. Three (10%) donors had more than one comorbid condition. There were 21 (70%) donors with no previous comorbid conditions. The donors who had hypertension did not require an increase in the dose of antihypertensives after donor nephrectomy.

**Table 1:** Baseline demographic data of the donors BMI: body mass index, BSA: body surface area, SBP: systolic blood pressure, DBP: diastolic blood pressure, SD: standard deviation

Variable	Mean	Range	SD
Age (years)	49.23	23-73	±12.29
Weight (kg)	60.72	43-96	±11.27
Height (cm)	157.2	147-170	± 5.8
BMI (kg/m^2^)	24.73	17.3-39.2	±5.58
BSA (m^2^)	1.59	1.39-1.85	±0.12
SBP (mmHg)	122.47	116.68-126.02	±9.07
DBP (mmHg)	80.47	78.77-81.76	±4.05

Pre-nephrectomy studies

The mean serum creatinine of all donors was 0.78 ± 0.12 mg/dL. The mean serum creatinine of male and female donors was 0.92 ± 0.19 mg/dL and 0.74 ± 0.22 mg/dL, respectively. Pre-nephrectomy, the mean measured total GFR was 103.83 ± 10.07 mL/minute/1.73 m^2^. In our study, we found that the measured GFR was between 80 and 90 mL/minute/1.73 m^2^ in six donors and >90 mL/minute/1.73 m^2^ in 24 donors. Pre-donation, all donors had a mean SBP of 122.47 ± 9.07 mmHg and DBP of 80.47 ± 4.05 mmHg (Table 1).

Post-nephrectomy studies

Three months post-donation, the mean serum creatinine of all donors was 1.03 ± 0.26 mg/dL. The mean serum creatinine of male and female donors was 1.21 ± 0.15 mg/dL and 0.98 ± 0.9 mg/dL, respectively. Three months after kidney donation, the mean measured GFR for all donors was 60.47 ± 6.57 mL/minute/1.73 m^2^. Post-donation, at three months, all donors had a mean SBP of 124.87 ± 10.64 mmHg and DBP of 81 ± 4.22 mmHg, respectively.

Pre- versus post-nephrectomy analysis

**Figure 1:**
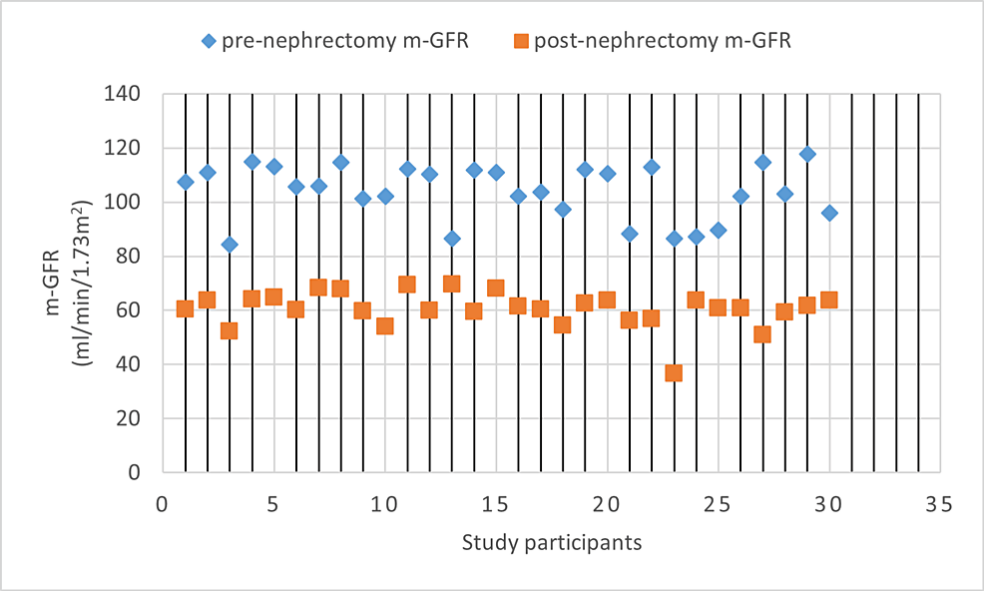
m-GFR pre- and post-nephrectomy x-axis: study participants (N = 30), y-axis: m-GFR pre- and post-nephrectomy m-GFR: measured glomerular filtration rate

Parameters including pre- and post-donation blood pressures, serum creatinine, and measured GFR were analyzed. As expected, there was a significant rise in serum creatinine of all donors from 0.78 mg/dL ± 0.12 mg/dL to 1.03 ± 0.26 mg/dL (p < 0.001). Also, the pre-nephrectomy total mean measured GFR of 103.83 mL/minute/1.73 m^2^ significantly dropped to 60.47 mL/minute/1.73 m^2^ three months post-nephrectomy (p < 0.001) (Table 2). A drop of 41.77% was seen in the total measured GFR three months after donation (Figure 1).

**Table 2:** Donor serum creatinine and m-GFR pre- and post-kidney donation m-GFR: measured GFR by Tc-99m DTPA scan, GFR: glomerular filtration rate, DTPA: diethylene triamine pentaacetic acid, SD: standard deviation

Variables	Pre-nephrectomy (mean ± SD)	Post-nephrectomy (mean ± SD)	p-value
Serum creatinine (mg/dL)	0.78 ± 0.12	1.03 ± 0.26	<0.001
m-GFR (mL/minute/1.73 m^2^)	103.8 ± 10.07	60.47 ± 6.57	<0.001

Changes in the GFR of the remnant kidney post-donation

Out of 30 donors, 28 (93.33%) had a compensatory increase in the measured GFR of the remnant kidney, while two (6.66%) had a fall in the measured GFR of the remnant kidney. In 28 donors who had an increased compensatory response post-donation, the remnant kidney had a mean increase in the measured GFR of 9.21 ± 4.39 ranging from 0.7 to 18.7 mL/minute/1.73 m^2^. Thirteen (43.33%) donors had a mean rise in m-GFR of more than 10 mL/minute/1.73 m^2^, 11 (36.66%) had a rise between 5 and 10 mL/minute/1.73 m^2^, and four (13.33%) had a rise between 0 and 5 mL/minute/1.73 m^2^. In two donors, there was a drop in the measured GFR of the remnant kidney by a mean of 6.8 ± 1.69 mL/minute/1.73 m^2^.

Rise in GFR in relation to age

**Figure 2:**
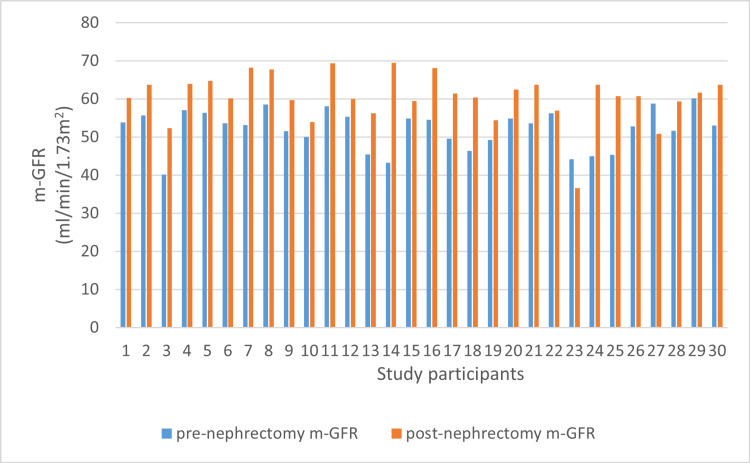
Rise in m-GFR in relation to age x-axis: study participants (N = 30), y-axis: m-GFR rise in relation to age m-GFR: measured glomerular filtration rate

In donors with comorbidities and age more than 50 years, the mean rise in the measured GFR was 8.63 ± 3.09 mL/minute/1.73 m^2^ ranging from 4 to 15.1 mL/minute/1.73 m^2^, while in those with age less than 50 years and no comorbidities, it was 9.72 ± 5.34 ranging from 0.7 to 18.7 mL/minute/1.73 m^2^. However, the rise in the measured GFR in both groups was statistically insignificant (p = 0.524) (Figure 2).

## Discussion

In the Indian scenario, there always has been an inclination toward live related kidney transplants rather than deceased donor kidney transplants [13]. To bridge the gap between organ shortage and increasing demand, donors with comorbidities and an older age group are also being considered as prospective kidney donors [14].

With aging, the decline in GFR is inevitable and is ecumenically associated with structural changes in the kidney. The proportion of glomeruli developing glomerulosclerosis increases steadily as age progresses, which leads to a gradual drop in GFR over the years [15]. Creatinine measurement is influenced by factors such as non-steady-state conditions and non-GFR determinants, which include diet, muscle mass, extrarenal elimination, and tubular secretion [16]. Studies have reported inaccuracies in GFR estimation with the use of serum creatinine-based GFR estimating equations in individuals with solitary kidneys due to congenital renal disorders or individuals who have undergone unilateral nephrectomy for living kidney donation or kidney diseases [17,18]. After nephrectomy, it is expected that the retained kidney undergoes compensatory changes, and its total GFR increases up to 70% of the preoperative range within 8-12 weeks of donation [19]. Keeping in mind the accelerated loss in total GFR after nephrectomy, it is imperative to predict the renal function of the retained kidney after kidney donation and determine the adequacy of compensatory abilities of the remnant kidney after donor nephrectomy. Thus, an accurate measurement of the GFR of kidney donors after donor nephrectomy forms an integral part of donor health and well-being.

In this study, the donor demographics, clinical parameters, and the changes in the measured GFR pre- and post-nephrectomy were studied. We observed that the pre-donation mean serum creatinine of all donors was 0.78 ± 0.12 mg/dL, which increased to 1.03 ± 0.26 mg/dL three months post-donation. In their study, Bahirani et al. [20] recorded a pre- and three-month post-donation mean serum creatinine of 0.81 ± 0.11 mg/dL and 1.01 ± 0.12 mg/dL, respectively. In terms of measured GFR by Tc-99m DTPA scan, the average pre-donation mean measured GFR for our population was 103.83 ± 10.07 mL/minute/1.73 m^2 ^as compared to the mean measured GFR of 99.47 ± 14.4 mL/minute/1.73 m^2^ and 112.8 mL/minute/1.73 m^2^ in studies conducted by Bahirani et al. [20] and Lin et al. [21], respectively. In a study by Mahajan et al. [22], they observed a mean GFR of 83.85 mL/minute/1.73 m^2^ using the DTPA clearance method, which was lower as compared to our study. We observed that three months after donation, the mean measured GFR was 60.47 ± 6.57 mL/minute/1.73 m^2^. This was comparable to a study by Bahirani et al. [20], where the mean GFR was 62.1 ± 11.5 mL/minute/1.73 m^2^ by Tc-99m DTPA scan when measured 90 days post-donation. As expected, there was a drop in the total measured GFR by 41% three months post-donation in our study.

Twenty-eight (93.33%) donors had a compensatory increase in remnant kidney GFR three months after donation. Among these donors, 13 (46.42%) had an age of less than 50 years, while 15 (53.57%) belonged to a group with an age of more than 50 years and comorbidities. The baseline mean measured GFR of the remnant kidney of these donors was 51.36 ± 5.54 mL/minute/1.73 m^2^(range: 52.3-69.5 mL/minute/1.73 m^2^), which increased to 61.67 ± 4.52 mL/minute/1.73 m^2^ (range: 52.3-69.5 mL/minute/1.73 m^2^) three months post-kidney donation, representing a 16.7% rise in the measured GFR of the remnant kidney. The mean measured GFR of the remnant kidney increased by 9.21 ± 4.39 mL/minute/1.73 m^2^. The increase in m-GFR in our study was lower as compared to studies by Chen et al. [23] and Bahirani et al. [20], who found a 22% and 21.2% increase, respectively, in remnant kidney GFR three months post-nephrectomy. Among the two donors who had a drop in m-GFR, the first donor was a 64-year-old male who had a drop of 8 mL/minute/1.73 m^2 ^with a serum creatinine of 1.8 mg/dL, and the second donor was a 56-year-old female, who had a drop of 5.6 mL/minute/1.73 m^2^ with a serum creatinine of 2.1 mg/dL and had developed acute pyelonephritis. Both donors had no known previous comorbidities and were diagnosed with hypertension after nephrectomy and started on a single antihypertensive. Older age group, the presence of pyelonephritis, and new-onset hypertension might have contributed to the drop in the GFR of the remnant kidney post-donation. In the study done by Lam et al. [24], older age groups, lower pre-donation estimated GFR, and pre-donation hypertension were associated with a greater decline in GFR one year post-donation.

In donors with a good compensatory response post-donation, we analyzed the mean rise in the measured GFR three months after nephrectomy in donors aged more than 50 years compared to donors aged less than 50 years and found that the increase in the measured GFR in this age group had no significant difference (9.72 ± 5.34 mL/minute/1.73 m^2^ versus 8.63 ± 3.09 mL/minute/1.73 m^2^, p = 0.524). This highlights the fact that despite the inclusion of donors with greater age and comorbidities, the compensatory rise in the measured GFR three months after donor nephrectomy was equivalent to young donors and donors without any comorbidities.

Our study limitations comprised a limited sample size, a short follow-up period, and a single-center study. We did not measure GFR using a gold standard method such as inulin clearance. Blood pressure measurements were not taken using 24-hour ambulatory BP monitoring. Further studies are required to determine which variables will predict if the donor will have a rise or fall in GFR after donation and compensatory changes in the remnant kidney post-donation.

## Conclusions

Previous studies on kidney donors have checked long-term outcomes following donor nephrectomy, but there have been very few studies assessing the changes in measured GFR post-donation and analyzing their sufficiency in the short term. Also, this study showed that the compensatory changes in remnant kidneys after nephrectomy at three months in donors with comorbidities and an older age group were comparable with donors aged less than 50 years.

Inaccurate and less frequent monitoring of GFR after kidney donation can have a negative impact on clinical decision-making and the health of individuals after kidney donation. To safeguard the health of kidney donors and reduce the risk of end-stage kidney disease in donors, there should be accurate measurement of GFR at various timelines after kidney donation. Long-term studies are necessary to study the changes in GFR at various timelines after kidney donation and determine the adequacy of compensatory changes in the remnant kidney.
